# Development and bin mapping of a Rosaceae Conserved Ortholog Set (COS) of markers

**DOI:** 10.1186/1471-2164-10-562

**Published:** 2009-11-29

**Authors:** Antonio Cabrera, Alex Kozik, Werner Howad, Pere Arus, Amy F Iezzoni, Esther van der Knaap

**Affiliations:** 1 Department of Horticulture and Crop Science, The Ohio State University/Ohio Agricultural Research and Development Center, Wooster OH 44691, USA; 2 Genome Center and Department of Plant Sciences, University of California, Davis, California 95616, USA; 3 Departament de Genètica Vegetal, Laboratori de Genètica Molecular Vegetal, CSIC-IRTA, 08348 Cabrils, Spain; 4 Department of Horticulture, Michigan State University, East Lansing MI 48824, USA

## Abstract

**Background:**

Detailed comparative genome analyses within the economically important Rosaceae family have not been conducted. This is largely due to the lack of conserved gene-based molecular markers that are transferable among the important crop genera within the family [e.g. *Malus *(apple), *Fragaria *(strawberry), and *Prunus *(peach, cherry, apricot and almond)]. The lack of molecular markers and comparative whole genome sequence analysis for this family severely hampers crop improvement efforts as well as QTL confirmation and validation studies.

**Results:**

We identified a set of 3,818 rosaceaous unigenes comprised of two or more ESTs that correspond to single copy Arabidopsis genes. From this Rosaceae Conserved Orthologous Set (RosCOS), 1039 were selected from which 857 were used for the development of intron-flanking primers and allele amplification. This led to successful amplification and subsequent mapping of 613 RosCOS onto the *Prunus *TxE reference map resulting in a genome-wide coverage of 0.67 to 1.06 gene-based markers per cM per linkage group. Furthermore, the RosCOS primers showed amplification success rates from 23 to 100% across the family indicating that a substantial part of the RosCOS primers can be directly employed in other less studied rosaceaous crops. Comparisons of the genetic map positions of the RosCOS with the physical locations of the orthologs in the *Populus trichocarpa *genome identified regions of colinearity between the genomes of *Prunus*-Rosaceae and *Populus*-Salicaceae.

**Conclusion:**

Conserved orthologous genes are extremely useful for the analysis of genome evolution among closely and distantly related species. The results presented in this study demonstrate the considerable potential of the mapped *Prunus *RosCOS for genome-wide marker employment and comparative whole genome studies within the Rosaceae family. Moreover, these markers will also function as useful anchor points for the genome sequencing efforts currently ongoing in this family as well as for comparative QTL analyses.

## Background

The Rosaceae is an important plant family that includes more than 90 genera and 3000 species. The family belongs to the Rosid clade and is closely related to the Salicaceae (including poplar), Leguminoseae (including *Medicago *and soybean), Cucurbitaceae (including cucumber and melon) and more distantly related to the Brassicaceae (including Arabidopsis). The Rosaceae is divided into three subfamilies, two of which include some of the most economically important temperate fruit crops [1]. The largest subfamily is the Spiraoideae to which *Malus *(apple), *Pyrus *(pear) and *Prunus *(peach, cherry, almond, apricot) belong. The second largest subfamily is the Rosoideae to which *Fragaria *(strawberry), *Rubus *(currants, blackberries, raspberries) and *Rosa *(rose) belong. Within the family, apple, peach and strawberry have been utilized as model species for Rosaceae biology, genetics and genomics [2].

Comparative analyses of plant genomes offer insights into genome evolution and speciation of closely as well as more distantly related species. In particular, knowledge of the extent and locations of syntenic blocks and chromosomal rearrangements enables the transfer of genomic information among species. This information would aid genome-wide as well as targeted marker development for the identification and validation of loci controlling traits that are important for crop improvement. Without the availability of several sequenced plant genomes within one family, comparative analyses often rely on molecular markers that are shared among the species. One of the earliest efforts towards the construction of comparative plant maps using molecular markers was conducted in the Solanaceae family. Assessment of the degree of similarity between tomato and pepper [3,4] and tomato and potato [5] show that the more closely related species, tomato and potato, underwent fewer rearrangements compared to the more distantly related tomato and pepper. Similarly in the Poaceae family, conservation of large chromosomal regions between wheat, barley and rye genomes have been identified [6,7]. The application of comparative sequence analysis within the grasses greatly facilitated the positional cloning of important genes such as *VRN1 *from wheat, a species for which map-based cloning was deemed impossible due to its large genome size and the presence of many repetitive elements that would hamper chromosome walking efforts [8].

Despite the lack of extensive investigations, the potential for comparative genome analysis within the Rosaceae family has been demonstrated by several studies. Genome colinearity was found among *Prunus *species [9-16]. These comparative studies were based on the *Prunus *reference map (x = 8), the most detailed genetic map in the Rosaceae, that is derived from an interspecific almond (*P. dulcis*) cv. Texas × peach (*P. persica*) cv. Earlygold (abbreviation TxE) F_2 _mapping population [10]. Good colinearity and marker transferability within the family was also demonstrated by the identification of syntenic regions of the *Malus *and *Prunus *genomes [9,17], and between the more distant genera *Prunus *and *Fragaria *[18,19]. However, a comprehensive and extensive comparative map such as those that were constructed in the Solanaceae and Poaceae families has not been achieved for the Rosaceae. This is mostly due to the lack of conserved markers to apply across the entire family [12,18].

Genes that are highly conserved and are present as low or single copy in genomes are particularly useful as markers for genome evolution studies as well as whole genome comparative analyses [20,17]. A Conserved Ortholog Set (COS) is defined as a collection of genes that are conserved in sequence and copy number throughout plant evolution [20]. In contrast, paralogs represent duplicated regions within the genome as a result of single gene duplications and/or large scale polyploidization events [21]. The development of markers from single copy and conserved genes is critical in comparative mapping studies as these markers enable an unambiguous determination of the degree of synteny [22]. In addition, the single copy conserved genes reduce the possibility of erroneously identifying chromosomal rearrangements that could result from mapping paralogous genes [23].

Complete whole-genome sequence information of model plants together with improved genomic resources from other species, such as EST databases, provide the opportunity for the *in silico *identification of candidate COS. Using the Arabidopsis whole genome sequence and the EST databases of potato, tomato and pepper, Wu et al identified 2869 Solanaceaous COS [21]. Likewise, a universal set of COS markers was developed for the Asteraceae family after comparing EST from sunflower and lettuce against the whole genome of Arabidopsis [24]. Moreover, comparative genome sequence analysis between the three sequenced model species, *Arabidopsis thaliana*, *Oryza sativa *and *Populus trichocarpa *resulted in the identification of 753 COS candidates among the angiosperms of which 55 to 359 could be identified from pairwise comparisons among four gymnosperm EST databases [25]. Once developed, COS markers have been widely employed to link the genomes of related species within families [20,26-29]

In this study, we report the first step towards a comprehensive and dense comparative genetic map for rosaceous species. We present the development of a set of conserved Rosaceae gene-based sequences corresponding to single copy Arabidopsis genes. These Rosaceae COS (RosCOS) were subsequently mapped using the bin map population corresponding to the *Prunus *TxE reference map [10,30]. Our analyses show that nearly all of the mapped RosCOS are present once in the *Prunus *genome suggesting that this genus did not undergo a hitherto unknown recent polyploidization event. Additionally, we compared the genetic location of these RosCOS to the physical location of the poplar and Arabidopsis orthologs. These analyses identified many regions that exhibited synteny between *Prunus *and poplar and to a lesser extent to Arabidopsis.

## Results and Discussion

### Construction of the RosCOS set

**Figure 1:**
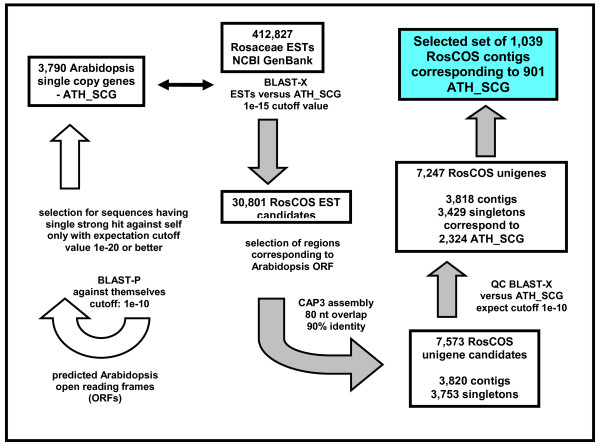
**Identification of Conserved Orthologous Set (COS) of sequences between Arabidopsis and Rosaceae**.

**Figure 2:**
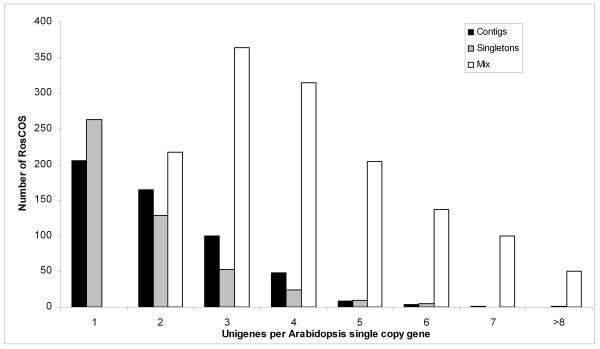
**Rosaceae unigene content per Arabidopsis single copy gene**. Numbers on the X-axis represent the number of Rosaceae unigenes matching a unique Arabidopsis single copy gene. Black bars represent unigenes comprised of at least two ESTs (contig); the gray bars represent singletons and white bars represent mixtures of singletons and contigs.

The Rosaceae ESTs that were publicly available as of December 2007 were used to construct the set of COS. The highest numbers of available Rosaceae ESTs were from *Malus*, *Prunus *and *Fragaria*, totaling up to 97.6% of all Rosaceae ESTs (Table 1). After comparing these ESTs to Arabidopsis single copy genes, we identified 30,801 putative orthologs (Figure 1). The CAP3 assembly of these ESTs resulted in 7,247 unigenes corresponding to 2,324 single copy Arabidopsis genes. Of these, 3,818 were contigs comprised of at least two ESTs and 3,429 were singletons. On average, the number of unigenes corresponded to 3.1 Rosaceae putative COS per Arabidopsis single copy gene. When we compared the distribution among contigs versus the singletons and the mixture of contigs and singletons, the majority of Arabidopsis single copy genes was represented by up to three Rosaceae unigenes (Figure 2). Also, the data showed that a significant number of the Arabidopsis single copy genes were represented by singletons indicating the lack of sufficiently deep EST data in the Rosaceae to permit assembly into contigs. The apparent redundancy in this unigene dataset is likely due to: 1) ESTs corresponding to the same gene but aligning to different parts of the gene, 2) sufficient nucleotide divergence within the Rosaceae EST from different species such that CAP3 would not allow them to be assembled into the same unigene, 3) errors in cloning, sequencing, as well as alternative splicing. Further investigation into the unigene duplicates is provided below.

**Table 1:** Number of Rosaceae ESTs from different subfamilies and genera.

**Sub-family**^**1**^	Genus	Total EST	EST corresponding to Arabidopsis COS
Rosoideae	*Fragaria*	50,882	3,899
Spiraeoideae	*Malus*	260,594	20,502
Spiraeoideae	*Prunus*	91,354	5,668

Spiraeoideae	*Pyrus*	341	8
Rosoideae	*Rosa*	9,289	712
Rosoideae	*Rubus*	323	12
Spiraeoideae	*Photinia*	44	0

	**Total**	**412,827**	**30,801**

^1^Classification from Potter et al [1].

Due to single pass sequencing of EST clones, the chance of sequencing errors can be considerable. In an effort to avoid the design of primers in regions of poor sequence quality, we focused on the 3,818 unigenes that were represented by at least two ESTs. Moreover, contigs tended to have more sequence information (i.e. longer sequences) which was helpful in the design of primers flanking the predicted intron sites. Each contig was named RosCOS### to indicate that this was the set of putatively conserved orthologous Rosaceae sequences. We narrowed the collection down further by selecting RosCOS that were represented by at least two of the three key genera in the family or *Prunus *alone (see Additional file 1). This selection was chosen to enhance the chance of successful amplification of *Prunus *DNA with the designed primers because of our goal to map these RosCOS on the *Prunus *reference map. The reduction led to the final data set of 1,039 RosCOS (Figure 1). We noticed that contigs harboring ESTs from more than one genus usually exhibited a higher number of mismatches in *Fragaria *than in *Malus *or *Prunus *which is consistent with the greater phylogenetic distance between *Fragaria *and the other two genera [1].

### Amplification and mapping of RosCOS in *Prunus*

**Figure 3:**
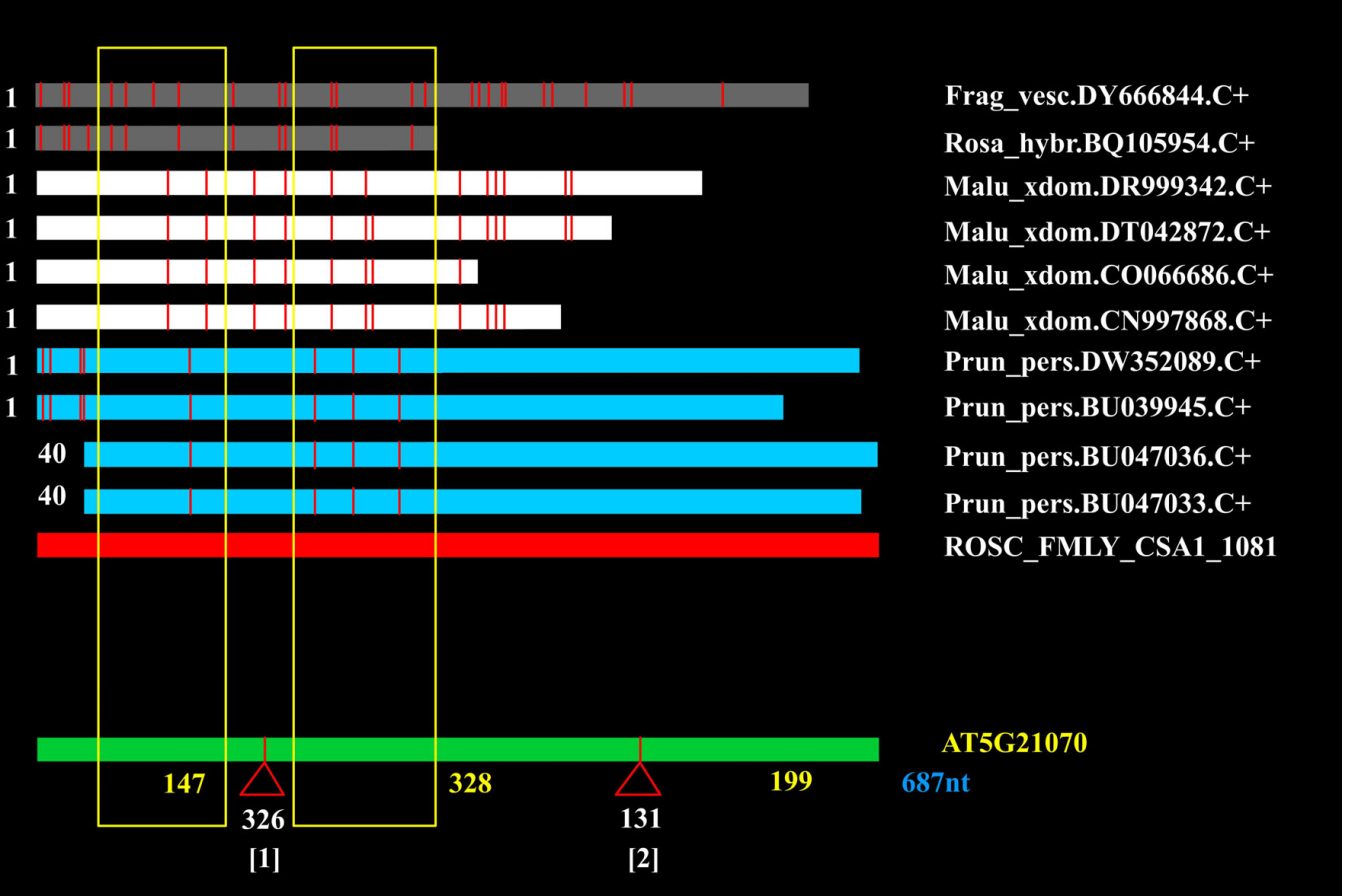
**Development of primers in adjacent exons that flank the same intron**. The output of the python contig software tool [40] allows the determination of the intron position based on the Arabidopsis genome sequence and EST constitution of RosCOS. The Rosaceae consensus sequence (red), composed of *Fragaria *(grey), *Malus *(white) and *Prunus *(blue) ESTs, and was compared to the Arabidopsis genome (green). The intron (Δ) and flanking positions (yellow blocks) were used to develop universal primers using Primer3 v. 0.4.0 [41].

Of the 1,039 RosCOS, 857 were selected for the design of intron-flanking primers because their sequences covered at least one putative intron (Figure 3). These primers were used to amplify the corresponding region from the TxE peach parent 'Earlygold', the F_1_, and the *Prunus *bin map set that consisted of six F_2 _individuals. Amplification success and mapping ability was evaluated, which demonstrated that 91% of the primers amplified *Prunus *DNA of which only 10% were monomorphic (Table 2). The percentage of RosCOS that exhibited only one SNP was 18% whereas 43% harbored at least 2 SNPs. A total of 39% of the polymorphic RosCOS contained at least one InDel (see Additional file 2 for detailed information about each RosCOS).

**Table 2:** Amplification and bin mapping success for 857 RosCOS primer pairs.

	Polymorphic RosCOS					
			
**Amplification in TxE**^**1**^	Bin mapped	**Orphan COS**^**2**^	**Putative bin 4:18**^**3**^	Inconclusive segregation	Failed sequence	Monomorphic
784 (91%)	607 (78%)	36 (5%)	6 (0.8%)	8 (1%)	47 (6%)	80 (10%)

^1^PCR amplification success in the TxE mapping population was visualized on agarose gels.

^2^RosCOS markers that mapped in bins that are not reported (orphan bins). Collectively, they represent 23 orphan bins of which 4 are comprised of two or more RosCOS.

^3^Heterozygous RosCOS in all the genotypes of the bin set resemble bin 4:18 on linkage group 4.

**Figure 4:**
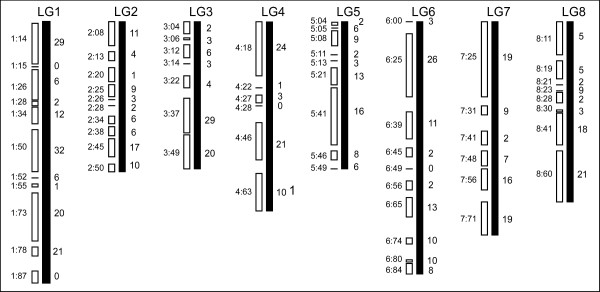
**Position of the 613 RosCOS on the TxE bin map**. Thick black vertical lines represent the linkage groups indicated above the lines. The white boxes on the left of each linkage group symbolize the bins (minimum bin length). The number before the semicolon indicates the linkage group and the number following the semicolon indicates the genetic position (in cM) of the last marker within the respective bin. Numbers on the right of each linkage group represent the number of RosCOS that map to the bin.

A total of 613 RosCOS were assigned to 63 of the 67 *Prunus *bins (Figure 4). This included six RosCOS for which the position could not be conclusively identified. For these six, the 'Earlygold' parent and F_1 _were both heterozygous as were all the F_2 _progeny individuals (Table 2). The heterozygous genotype found for all six F_2 _plants comprising the bin population is indicative of the position on the top of linkage group 4. Therefore, we tentatively placed these six RosCOS with the other RosCOS in bin 4:18 (Figure 4). However, it was also possible that these RosCOS represented recent gene duplications as was observed in a few other cases (Howad and Arus, unpubl). In addition, some RosCOS were clearly polymorphic but could not be assigned to an existing bin. The 36 unbinned RosCOS were termed "orphan COS" of which 17 grouped in four distinct bins. The fact that several orphan COS clustered together suggested that these bins correctly represent the *Prunus *genome; however, the genomic location is unknown. Only 1% of the RosCOS exhibited ambiguous segregation due to difficulty in scoring the SNPs and associated double peaks in the chromatograms. Six percent of the sequencing reactions failed, indicating the overall high quality of the sequence data (Table 2). In all, the average marker density per centimorgan (cM) ranged from 0.67 to 1.06 for the eight *Prunus *chromosomes (Table 3). Marker density within bins ranged from 0.2 to 18 per cM which might be indicative of regions of low and high recombination frequencies, respectively.

**Table 3:** RosCOS marker density on the eight *Prunus *TxE linkage groups.

Linkage Group	cM length of the linkage group	Number of RosCOS mapped	RosCOS density per cM
1	87.0	129	0.67
2	50.5	69	0.73
3	48.4	67	0.72
4	62.5	59	1.06
5	49.1	67	0.73
6	83.7	85	0.98
7	70.6	72	0.98
8	55.9	65	0.86

The marker density per linkage group is high and could be inflated due to the fact that one single copy Arabidopsis gene is represented by, on average, 3.1 Rosaceae unigenes (see above). Among the mapped RosCOS, we noted that 55 Arabidopsis single copy genes corresponded to two or more RosCOS (Table 4, see Additional file 3). Importantly, five of the 55 putatively duplicated Arabidopsis single copy genes mapped to different positions in the *Prunus *genome, indicating that at least some genes were duplicated in *Prunus *while they were single copy in Arabidopsis (Table 4). The remaining 50 putatively duplicated genes mapped to the same bin which implied that these could have been derived from one Rosaceae conserved gene (see Additional file 3). To further address this possibility, a closer examination of the CAP3 assembly of the 50 single copy Arabidopsis genes with more than one RosCOS representative revealed that in 36 cases these RosCOS corresponded to the same region of the Arabidopsis single copy gene. The reason that these unigenes were not assembled into one RosCOS appeared to stem from the fact that the overlapping region was too short and/or too divergent to ensure the assembling into one RosCOS. It is therefore likely that these RosCOS correspond to a single Rosaceae conserved gene and are not the result of gene duplication. For the remaining 14 putatively duplicated Arabidopsis single copy genes, the corresponding RosCOS did not overlap with the same region of the Arabidopsis gene. Therefore, whether these RosCOS corresponded to the same gene or a tandemly duplicated gene pair could not be determined with the present data. However, despite the evidence of a few duplicated genes, which may have occurred after the divergence of Arabidopsis-Brassicaceae and Rosaceae or represent gene loss in Arabidopsis, these data strongly support the evidence for the lack of a recent large scale genome duplication event in *Prunus*.

**Table 4:** Number of Arabidopsis single copy genes corresponding to more than one RosCOS and their *Prunus *bin map co-localization.

Number of Arabidopsis genes that correspond more than one RosCOS	RosCOS bin map locations		
	
	Map to the same bin	**Map to separate bins**^**1**^	Total
55	103 (91%)	10 (9%)	113 (100%)

^1^Five Arabidopsis single copy genes corresponded to two RosCOS that map to different positions in *Prunus*, indicating possible *Prunus *gene duplications not found in Arabidopsis.

### Synteny between Rosaceae, Arabidopsis and *Populus*

**Figure 5:**
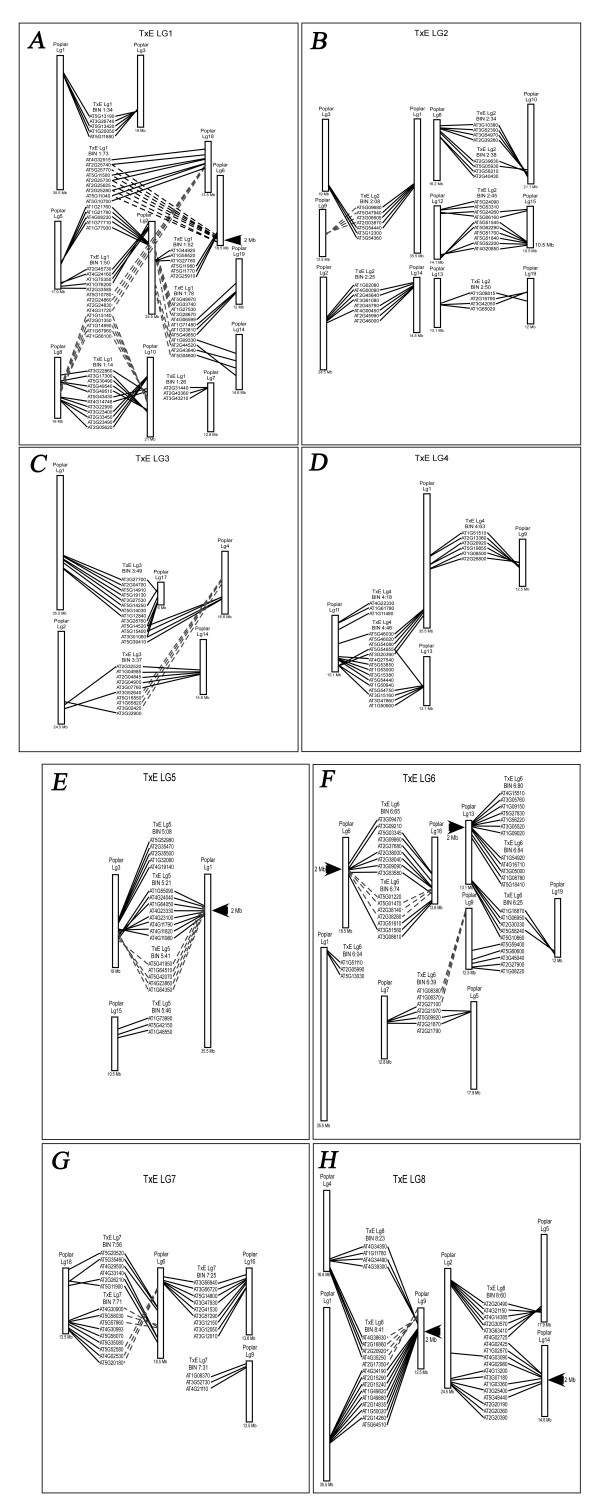
**Synteny between *Prunus *and *Populus***. Arabidopsis single copy genes corresponding to the bin mapped RosCOS were compared to the poplar genome. RosCOS that mapped to the same bin were selected for synteny analysis when three or more poplar orthologs were within 2 Mb from another for at least one poplar linkage group. Arrows indicate the largest syntenic blocks within 2 Mb of the poplar genome. A through H represent *Prunus *linkage groups 1 through 8, respectively.

The availability of the Arabidopsis and poplar genomes allowed us to determine the level of synteny among these species and *Prunus*. Because gene annotation is more complete for Arabidopsis than any other plant species, the translated Arabidopsis single copy genes corresponding to RosCOS that mapped to the same TxE bin were searched against the translated poplar genome using the TBLASTN function. After identifying the location in poplar of RosCOS that mapped together in *Prunus*, we found several syntenic regions between these genomes (Figure 5). Importantly, the mapping of the poplar COS confirmed nearly all the previously reported homeologous gene blocks shared by two poplar chromosomes presumed to have arisen from the most recent salicoid wide-genome duplication event [31]. For instance, RosCOS that mapped in the TxE bin 1:34 confirmed duplicated blocks of poplar linkage groups 1 and 3 (Figure 5A). The high level of synteny between poplar and *Prunus *as well as the conservation of gene order in paralogous regions of the poplar genome strongly supported the potential of the RosCOS for comparative mapping across the Rosaceae family. These results also suggested that the order of the RosCOS in the Rosaceae can be predicted based on their order in poplar, although this would have to be confirmed by genome sequence analysis or higher resolution genetic mapping. The size of the syntenic blocks was defined as large (more than seven RosCOS corresponding to poplar orthologs in a 2 Mb region), medium (harboring five to six RosCOS) and small (harboring three to four RosCOS). As a result, we identified six large syntenic blocks represented by bins 1:73, 8:41, 8:60, and the adjoining bins 5:21 and 5:41; 6:65 and 6:74; and 6:80 and 6:84. In addition, 21 medium and 20 small syntenic blocks were also observed (Figure 5).

**Figure 6:**
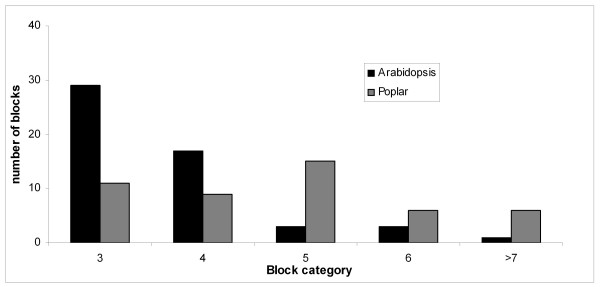
**Syntenic block size of *Prunus *with Arabidopsis and poplar**. Numbers on the X-axis represent the number of RosCOS that mapped to the same *Prunus *bin which were also identified within 2 Mb in the Arabidopsis and poplar genomes, black and gray bars, respectively.

We also analyzed the number of RosCOS that mapped to the same *Prunus *bin and their corresponding position in the Arabidopsis genome. The data indicated that the Arabidopsis -*Prunus *synteny blocks tended to be smaller compared to the size of the *Populus-Prunus *blocks (Figure 6). For example, most of the blocks in Arabidopsis had only three RosCOS within a 2 Mb interval whereas most of the blocks in poplar had five RosCOS within a 2 Mb interval. This result suggested that the order of the *Prunus *RosCOS is less conserved with that of Arabidopsis compared to poplar. This is an expected finding since Arabidopsis is more distantly related to Rosaceae than is poplar and is consistent with previous findings [32].

### Amplification of RosCOS across the Rosaceae

The transferability of molecular markers across different species is an important feature of conserved orthologous sequences in addition to the common ancestry these sequences represent. To explore the applicability of RosCOS markers in other rosaceous crops, a subset of the RosCOS primers was employed to amplify *Malus*, *Prunus *and *Fragaria *DNA. *Malus *and *Prunus *are phylogenetically closer than *Fragaria *as the former two belong to the same subfamily (Table 1). Despite the larger distance and the multiple SNP between the species, using EST information from all three genera enabled the development of primers that resulted in successful amplification of more than half of the RosCOS in each genus (Table 5). Amplification failures were likely due to the difficulty in designing primers with less than two mismatches in all three genera and presence of a large intron. The amplification success rate remained approximately the same when only two genera contributed to the RosCOS. However, when a RosCOS was represented by two genera, the lowest amplification was observed in the genus for which no EST contributed to the RosCOS. Yet, even when only *Prunus *EST information is used, the amplification success rate was 77% in *Malus *and 23% in *Fragaria*. In general, it is evident from these data that successful amplification across all genera is enhanced when EST from two genera contributed to the RosCOS and primer design.

**Table 5:** Amplification success of RosCOS primers in different genera.

Genera represented in each RosCOS	RosCOS per Group	Amplification in *Malus*	Amplification in *Fragaria*	Amplification in *Prunus *(cherry)
*Fragaria, Malus *and *Prunus*	7	7 (100%)	4 (57%)	4 (57%)
*Fragaria *and *Prunus*	13	8 (61%)	11 (85%)	10 (77%)
*Fragaria *and *Malus*	10	7 (70%)	10 (100%)	6 (60%)
*Prunus *and *Malus*	18	16 (89%)	9 (50%)	15 (83%)
*Prunus*	13	10 (77%)	3 (23%)	13 (100%)

## Conclusion

Comparative genome analysis for the Rosaceae family lags behind that of other economically important families such as the Solanaceae and Poaceae. The RosCOS resource developed in this study aims to ameliorate this situation by providing a marker set that can be employed for comparative mapping and marker development as well as whole genome comparative analyses in the Rosaceae family. The extensive colinearity observed between poplar and *Prunus *demonstrates the possibility of additional marker development in targeted regions of the *Prunus *genome based on synteny with poplar. Moreover, with the advent of Rosaceae species whole genome sequence information that will become available in the near future, these RosCOS will be instrumental to place unlinked scaffolds onto genetic maps and enable marker development to targeted regions in species whose genome is not sequenced. Excellent genetic maps and whole genome sequence data are extremely important for QTL discovery and validation. Therefore, the RosCOS resource developed herein has great potential to benefit rosaceaous crop improvement.

## Methods

### Identification of the Rosaceae COS (RosCOS) set

The set of 3,790 Arabidopsis single copy genes was selected as previously described [33]. The complete data set of 412,827 Rosaceae ESTs as of December 2007 was downloaded from NCBI GenBank [34] and compared to the Arabidopsis single copy gene set using the BLASTX function at the cutoff E-value of 1e-15. The resulting Rosaceae ESTs were assembled using the Contig Assembly Program: CAP3 [35] with parameters of at least 80 bp overlap and 90% sequence identity. This resulted in the assembly of 7,247 unigenes (3,818 contigs and 3,429 singletons) (Figure 1). The 3,818 contigs were assigned a RosCOS number whereas the singletons were not. The consensus sequence for each RosCOS is found under the name ROSC_FMLY_CSA1_1 beginning with RosCOS 1 [36]. The ESTs that are part of the RosCOS are found in the "December 2007 Assembly Info" [37]. The list of single copy Arabidopsis genes and corresponding RosCOS are found under the "December 2007 BLAST info" links [37]. Information about the final list of RosCOS used in this study can be found under the "RosCOS final selection and QC BLAST" links [37]. RosCOS map and primer data is also available from our own database [38] as well as in Additional file 2. Sequence data of the peach parent 'Earlygold' has been deposited in GSS at Genbank [34] and the corresponding accession numbers are listed in Additional file 2 (sheet 2).

### Design of PCR primers flanking introns

Orthologous genes share conserved structures such that the position of the introns is conserved [39]. To reduce the probability of sequencing errors in the ESTs and to increase the amplification success rate in multiple Rosaceae species, singletons were discarded from the analysis. Rosaceae contigs comprised of ESTs from three (*Fragaria*, *Malus *and *Prunus*) and two (*Fragaria*-*Malus, Prunus-Fragaria*, and *Malus*-*Prunus*) genera as well as only *Prunus *ESTs were selected totaling up to 1,039 RosCOS that were further investigated. The RosCOS were aligned to the Arabidopsis genome and putative intron sites were identified using the Python Contig Viewer program [40] (Figure 3). Based on the RosCOS sequence length and predicted intron position of these 1039 RosCOS, 857 intron-flanking primer pairs were developed using Primer3 v0.4.0 [41]. Subsequently, all forward primers were designed with an additional M13 tail (CACGACGTTGTAAAACGAC) at the 5' end to facilitate high-throughput direct sequencing of the amplicons.

### PCR conditions and polymorphism detection of RosCOS

The RosCOS putative intron-flanking primers were used to amplify the peach parent 'Earlygold', F_1 _and 6 bin set individuals selected from the *Prunus *TxE F_2 _reference population [10,30]. The amplification reactions were conducted in 96-well plate format in 60 ul reaction volume consisting of 10 mM Tris-Cl pH 8.3, 50 mM KCl, 2 mM MgCl_2_, 10-100 ng of genomic DNA, 0.1 mM of each dNTP, 0.1 uM of each primer, and 0.25 U Taq polymerase. The reactions were preheated at 94°C for 1 min followed by 31 cycles of 92°C (30 s), 56°C (30 s), 72°C (30 s), and a final extension of 72°C (60 s). Amplified fragments were sequenced using the M13F primer at the Agencourt Bioscience Corporation (Agencourt, Beverly, MA, USA). The sequencing results were analyzed for polymorphisms such as single nucleotide polymorphism (SNP) and/or insertion-deletions (InDels) using Sequencher software v4.2 (Gene Codes Corporation). The presence of a double peak in an otherwise high-quality chromatogram was indicative of the presence of a SNP. The sudden decay of high-quality chromatogram was indicative of the presence of an InDel.

### Genotyping and Mapping

Bins representing the different regions of the *Prunus *genome have been identified by the genotype of a subset of plants from the TxE F_2 _population [30]. RosCOS markers with a segregation pattern corresponding to a bin set score were grouped in that bin. RosCOS that mapped in bin 2:45 or 3:04 and 5:41 or 8:30, respectively, were analyzed in a 7^th ^genotype to map them in one or the other bin. RosCOS markers that clearly segregated but did not fall into a known bin were categorized as "orphan" RosCOS markers.

### Synteny of RosCOS and Poplar COS

The translated sequence of Arabidopsis single copy genes corresponding to the bin-mapped RosCOS were compared to the *Populus trichocarpa *genome. Using the *P. trichocarpa v1.1 *genome browser [42], the physical position of each poplar COS was identified through the TBLASTN function with the cut off E-value of 1e-5. Syntenic blocks between *Prunus *and poplar were established under the condition that a minimum of three linked RosCOS corresponded to poplar COS that were located within 2 Mb from each other.

## Authors' contributions

AC performed the intron-flanking primer design, the amplification reactions, the bin mapping experiments, the comparative analysis with poplar and Arabidopsis, and analyzed the data. AK developed the pipeline for the RosCOS identification. WH and PA provided the DNAs from the TxE bin set and critically evaluated the analysis of the bin mapping data. AI provided overall advice and coordination of this project, and participated in the supervision. EV conceived the RosCOS idea, designed and supervised the study. AC and EV wrote the manuscript with edits from the other coauthors. All authors read and approved the final manuscript.

## Supplementary Material

Additional file 1 Genus representation and primer development of RosCOS. Click here for file

Additional file 2 RosCOS marker, map, primer, SNP and InDel, accession number information. Click here for file

Additional file 3 **Map location of two or more RosCOS identified by one Arabidopsis single copy gene.** Dataset shows the map position of the putatively duplicated COS. Click here for file
