# Supplementary material for: Development and bin mapping of a Rosaceae Conserved Ortholog Set (COS) of markers
Source: BMC Genomics. 2009 Nov 29;10:562. doi: 10.1186/1471-2164-10-562 (PMC2789105; doi:10.1186/1471-2164-10-562)
Supplement: Additional file 1 — Genus representation and primer development of RosCOS. [file 1471-2164-10-562-S1.doc]

| **Additional file 1. Genus representation and primer development of RosCOS** | | |
| --- | --- | --- |
| **Genus** | **Number of RosCOS represented by a minimum of 2 EST** | **RosCOS primers developed^1^** |
| *Fragaria, Malus* and *Prunus* | 11 | 7 |
| *Fragaria* and *Prunus* | 14 | 13 |
| *Fragaria* and *Malus* | 10 | 10 |
| *Prunus* and *Malus* | 310 | 274 |
| *Prunus* only | 694 | 553 |
| Total | 1039 | 857 |
| ^1^Primers were only developed for RosCOS with a predicted intron and of sufficient length of the sequence. | | |
