# Supplementary material for: Development and bin mapping of a Rosaceae Conserved Ortholog Set (COS) of markers
Source: BMC Genomics. 2009 Nov 29;10:562. doi: 10.1186/1471-2164-10-562 (PMC2789105; doi:10.1186/1471-2164-10-562)
Supplement: Additional file 3 — Map location of two or more RosCOS identified by one Arabidopsis single copy gene. Dataset shows the map position of the putatively duplicated COS. [file 1471-2164-10-562-S3.doc]

**Additional file 3. Map location of two or more RosCOS identified by one Arabidopsis single copy gene.**

The linkage groups are indicated above the bin columns. RosCOS corresponding to the same Arabidopsis single copy gene are shown at the left for each bin. The numbers 2090/1523 correspond to the respective RosCOS###; the AT3G23400 correspond to the Arabidopsis single copy gene. Highlighted in paired colors or shades of gray are the 10 RosCOS corresponding to five Arabidopsis single copy genes that appear duplicated in the *Prunus* genome.
